# Supplementary material for: Optimization of In Situ Indentation Protocol to Map the Mechanical Properties of Articular Cartilage
Source: Materials (Basel). 2022 Sep 16;15(18):6425. doi: 10.3390/ma15186425 (PMC9505484; doi:10.3390/ma15186425)
Supplement: Supplementary file 1 [file materials-15-06425-s001.zip › materials-1861581-supplementary.pdf]

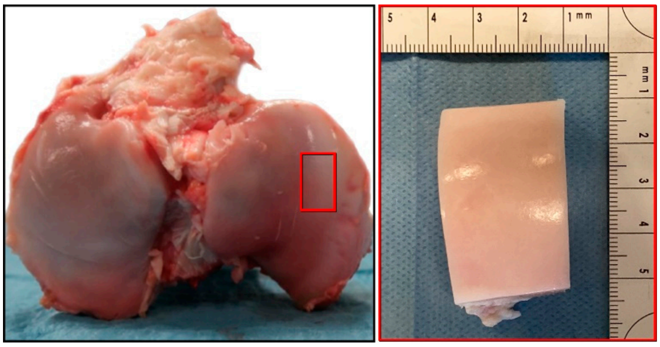

Supplementary Figure S1. Example of a bovine knee tibial plateau (left), from which samples suitable for indentation test were extracted (right).

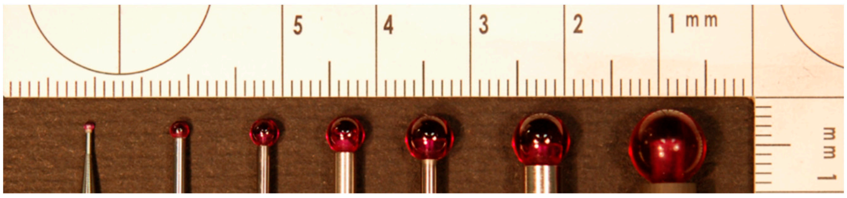

Supplementary Figure S2. Spherical indenters used to test knee articular cartilage, starting from 1-mm (left) to 8-mm (right) diameter indenter.

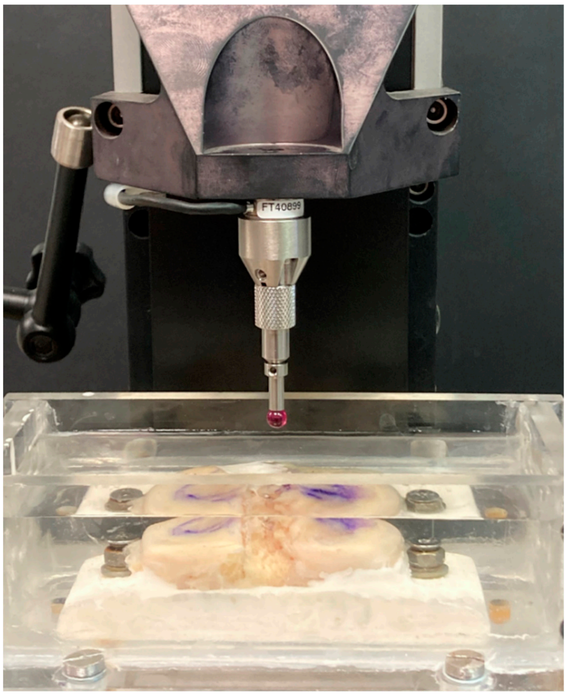

Supplementary Figure S3. The human tibial plateau constrained to the X-Y motorized table of the testing machine. The 6-mm diameter indenter is also visible.

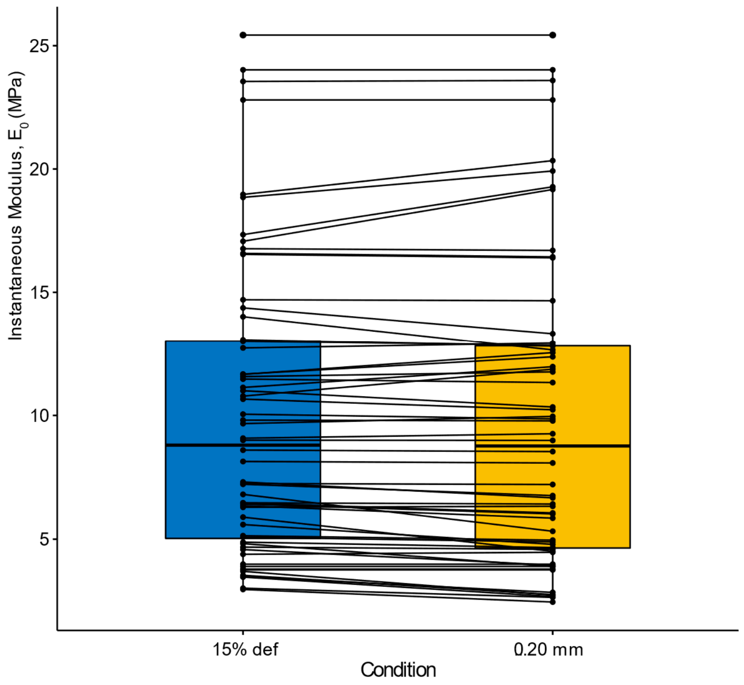

Supplementary Figure S4. Distributions of E_0 values determined with different imposed deformation, i.e., a nominal deformation of 15%, and a 0.20 mm displacement.

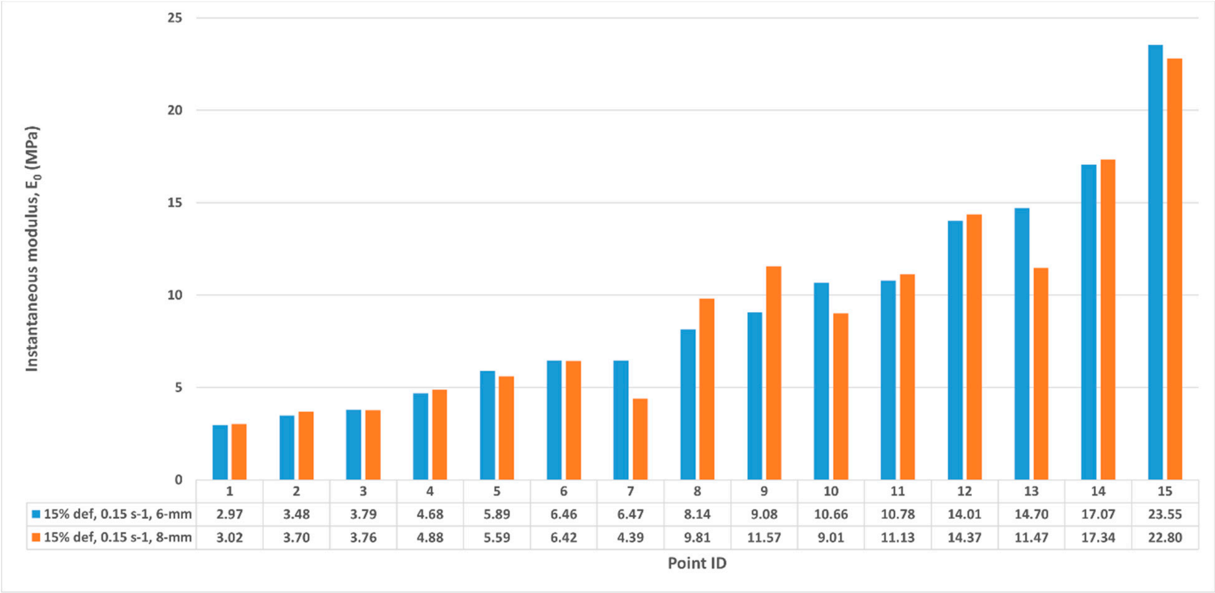

Supplementary Figure S5. E_0 values determined for the investigated points onto the human tibial plateau by using 6-mm and 8-mm indenters (nominal deformation of 15% of the cartilage thickness, 0.15 s^{-1} indentation rate).

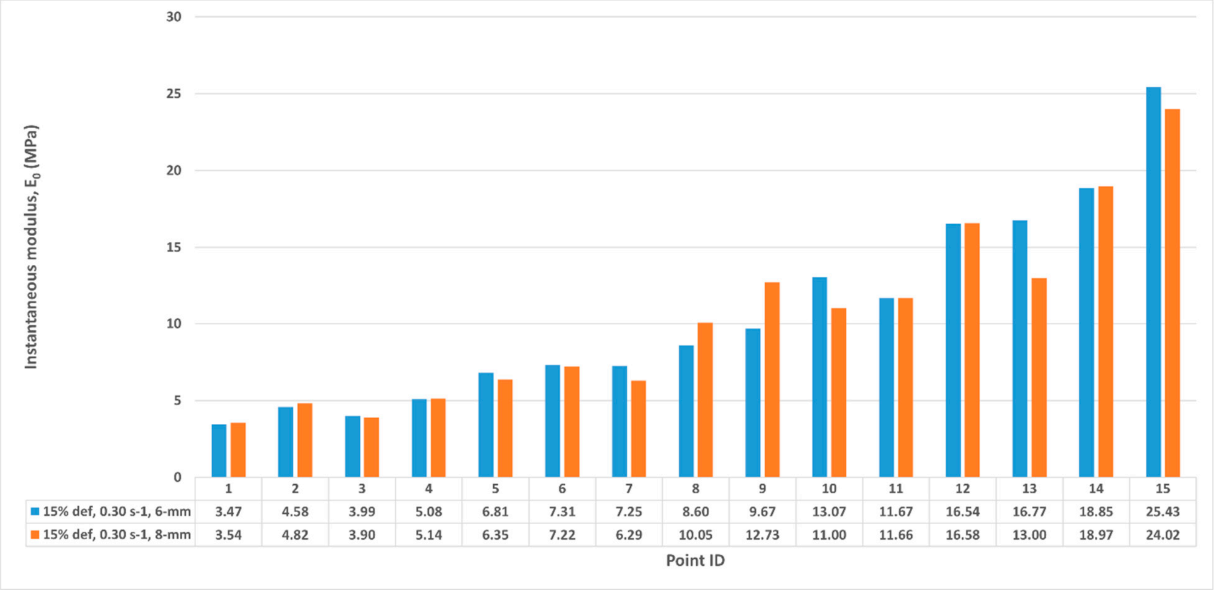

Supplementary Figure S6. E_0 values determined for the investigated points onto the human tibial plateau by using 6-mm and 8-mm indenters (nominal deformation of 15% of the cartilage thickness, 0.30 s^{-1} indentation rate).

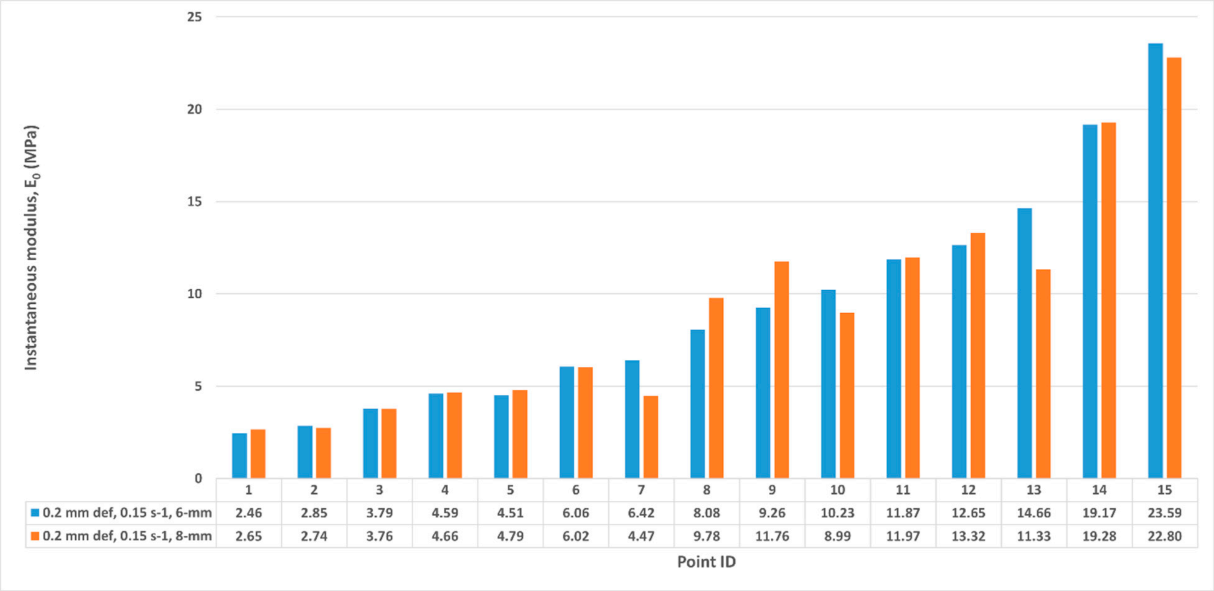

Supplementary Figure S7. E_0 values determined for the investigated points onto the human tibial plateau by using 6-mm and 8-mm indenters (indentation depth of 0.20 mm, 0.15 s^{-1} indentation rate).

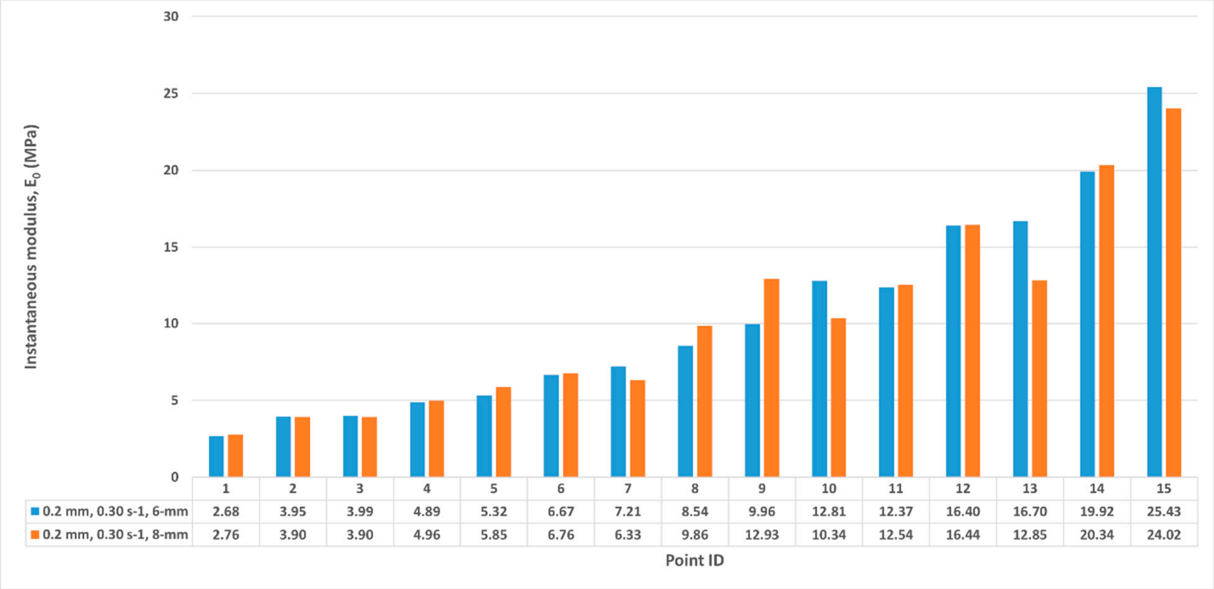

Supplementary Figure S8. E_0 values determined for the investigated points onto the human tibial plateau by using 6-mm and 8-mm indenters (indentation depth of 0.20 mm, 0.30 s^{-1} indentation rate).

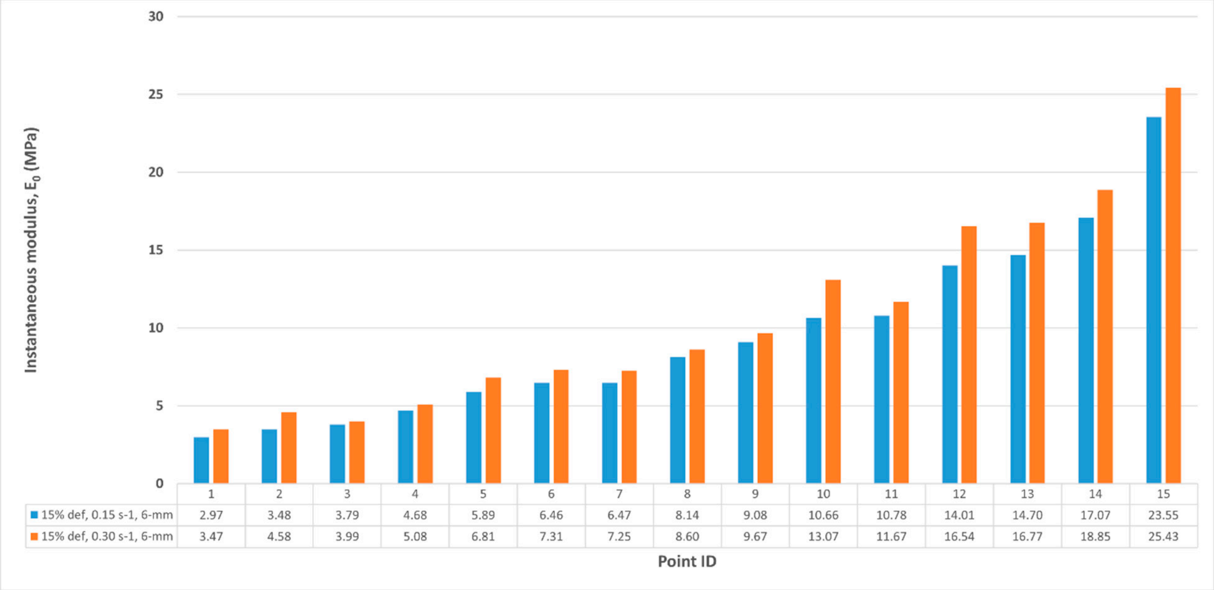

Supplementary Figure S9. E_0 values determined for the investigated points onto the human tibial plateau by using 0.15 s^{-1} and 0.30 s^{-1} indentation rate (nominal deformation of 15% of the cartilage thickness, 6-mm indenter diameter).

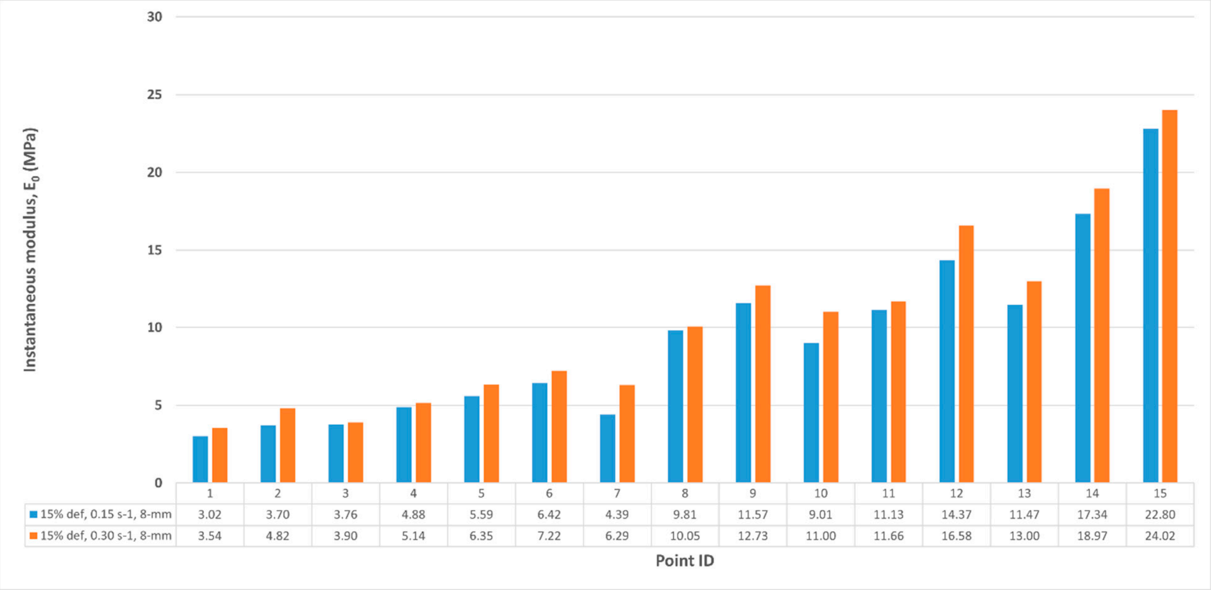

Supplementary Figure S10. E_0 values determined for the investigated points onto the human tibial plateau by using 0.15 s^{-1} and 0.30 s^{-1} indentation rate (nominal deformation of 15% of the cartilage thickness, 8-mm indenter diameter).

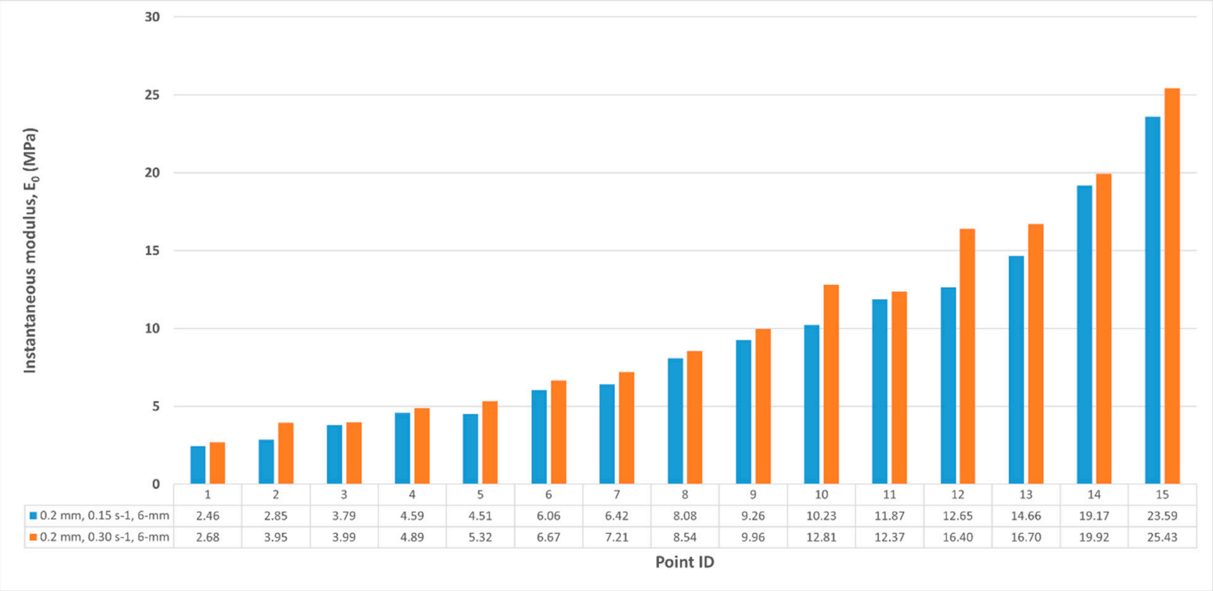

Supplementary Figure S11. E_0 values determined for the investigated points onto the human tibial plateau by using 0.15 s^{-1} and 0.30 s^{-1} indentation rate (indentation depth of 0.20 mm, 6-mm indenter diameter).

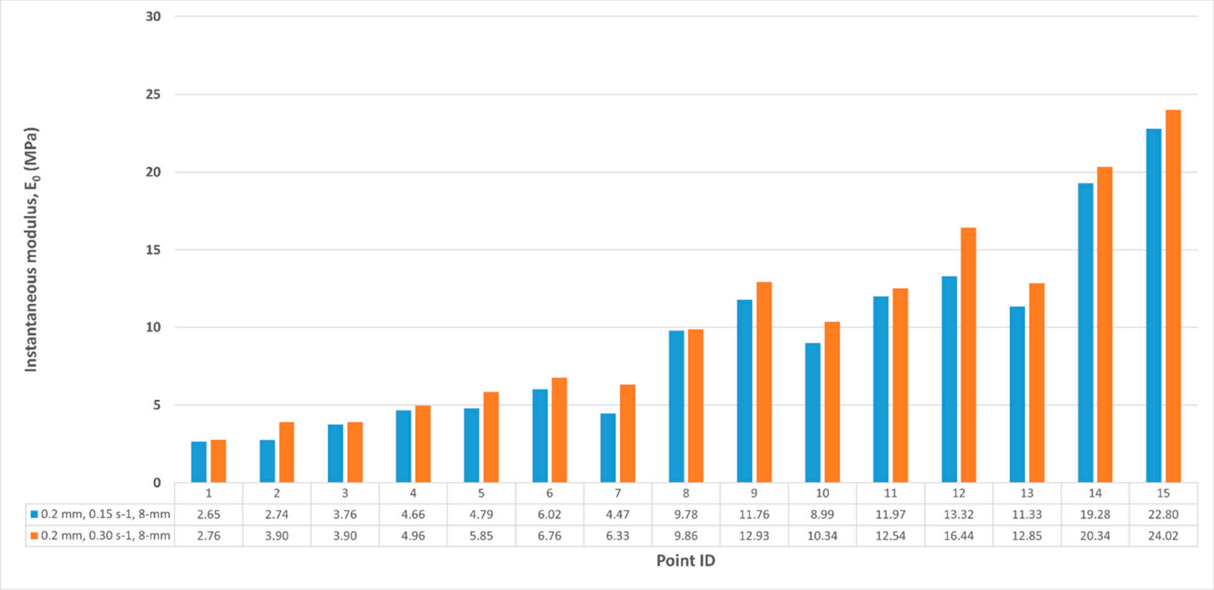

Supplementary Figure S12. E_0 values determined for the investigated points onto the human tibial plateau by using 0.15 s^{-1} and 0.30 s^{-1} indentation rate (indentation depth of 0.20 mm, 8-mm indenter diameter).

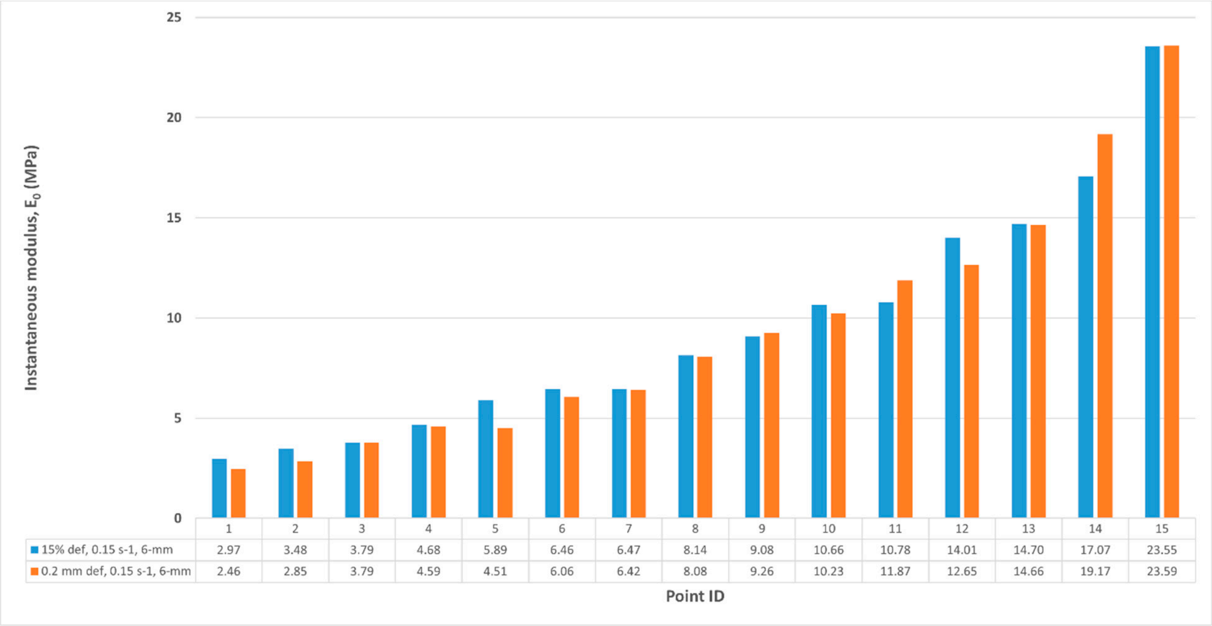

Supplementary Figure S13. E_0 values determined for the investigated points onto the human tibial plateau by applying a nominal deformation of 15% of the cartilage thickness, or an indentation depth of 0.20 mm (indentation rate of 0.15 s^{-1}, 6-mm indenter diameter).

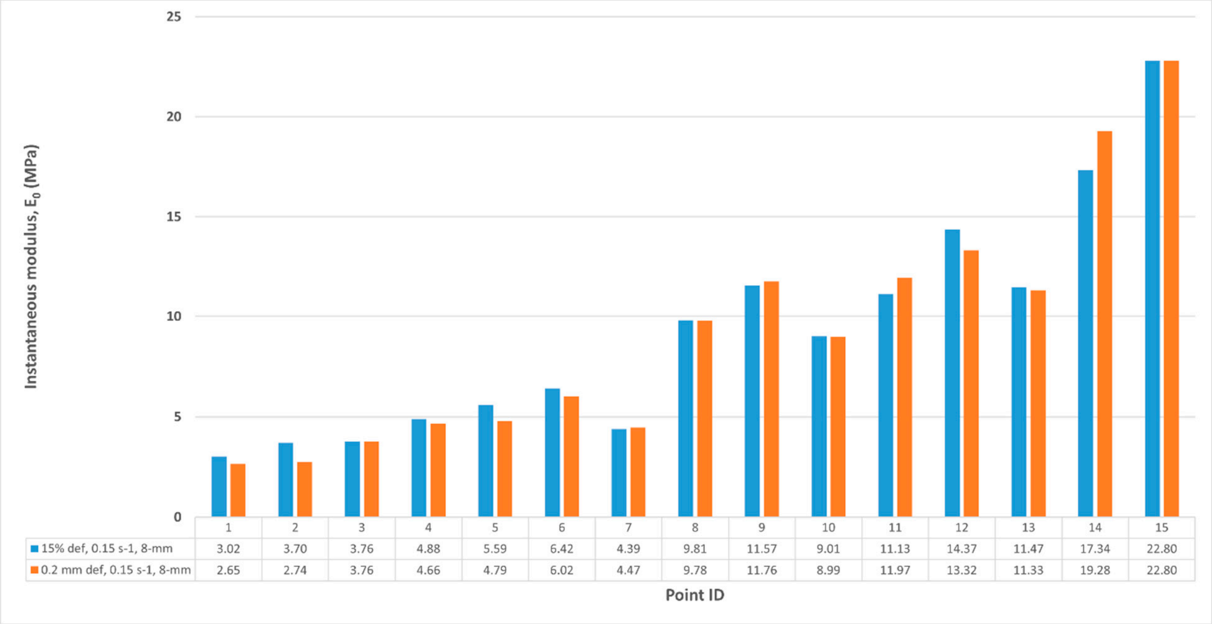

Supplementary Figure S14. E_0 values determined for the investigated points onto the human tibial plateau by applying a nominal deformation of 15% of the cartilage thickness, or an indentation depth of 0.20 mm (indentation rate of 0.15 s^{-1}, 8-mm indenter diameter).

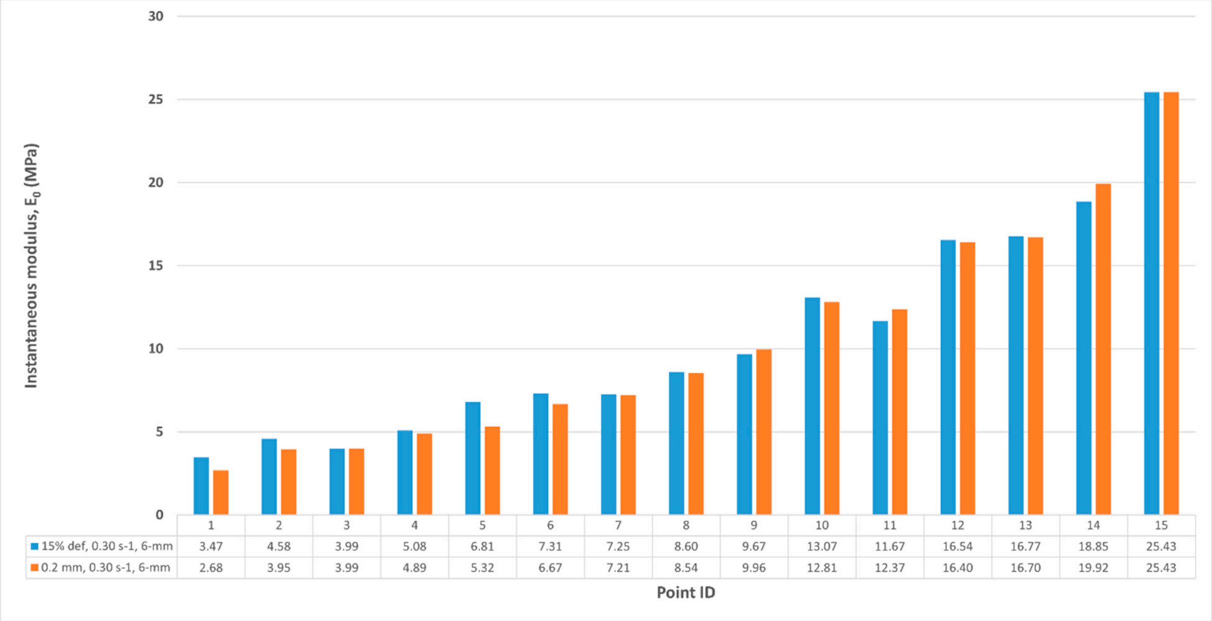

Supplementary Figure S15. E_0 values determined for the investigated points onto the human tibial plateau by applying a nominal deformation of 15% of the cartilage thickness, or an indentation depth of 0.20 mm (indentation rate of 0.30 s^{-1}, 6-mm indenter diameter).

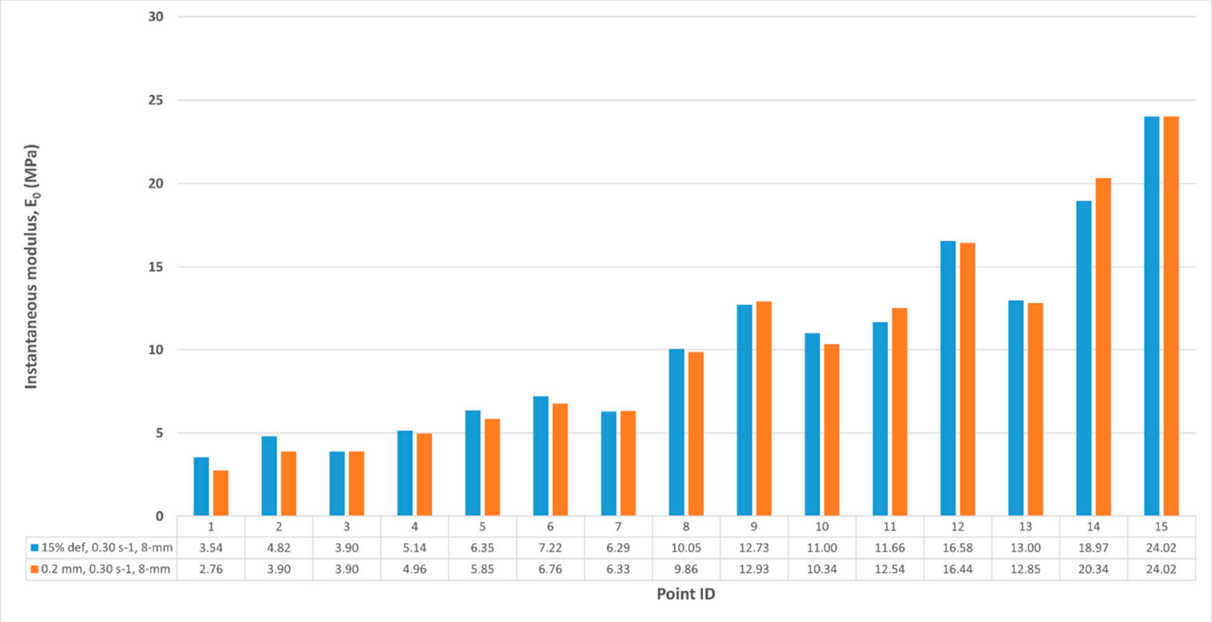

Supplementary Figure S16. E_0 values determined for the investigated points onto the human tibial plateau by applying a nominal deformation of 15% of the cartilage thickness, or an indentation depth of 0.20 mm (indentation rate of 0.30 s^{-1}, 8-mm indenter diameter).

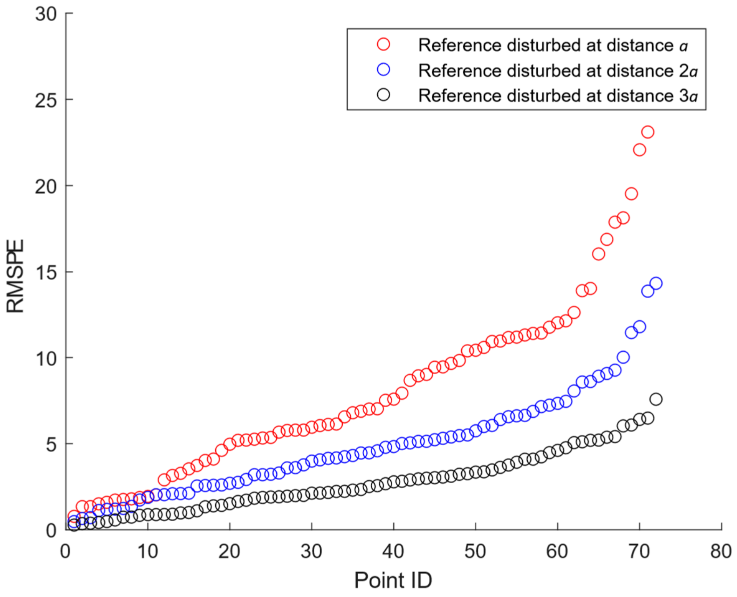

Supplementary Figure S17. Root mean square percentage error (RMSPE) calculated comparing the 72 stretched-exponential functions describing the response of the cartilage without perturbation (noPI) with each of the stretched-exponential function describing the response of the perturbed cartilage (PI*a*, PI2*a*, PI3*a*) in the same 72 measuring points.
