# Supplementary figures and images for: Optimization of In Situ Indentation Protocol to Map the Mechanical Properties of Articular Cartilage
Source: Materials (Basel). 2022 Sep 16;15(18):6425. doi: 10.3390/ma15186425 (PMC9505484; doi:10.3390/ma15186425)

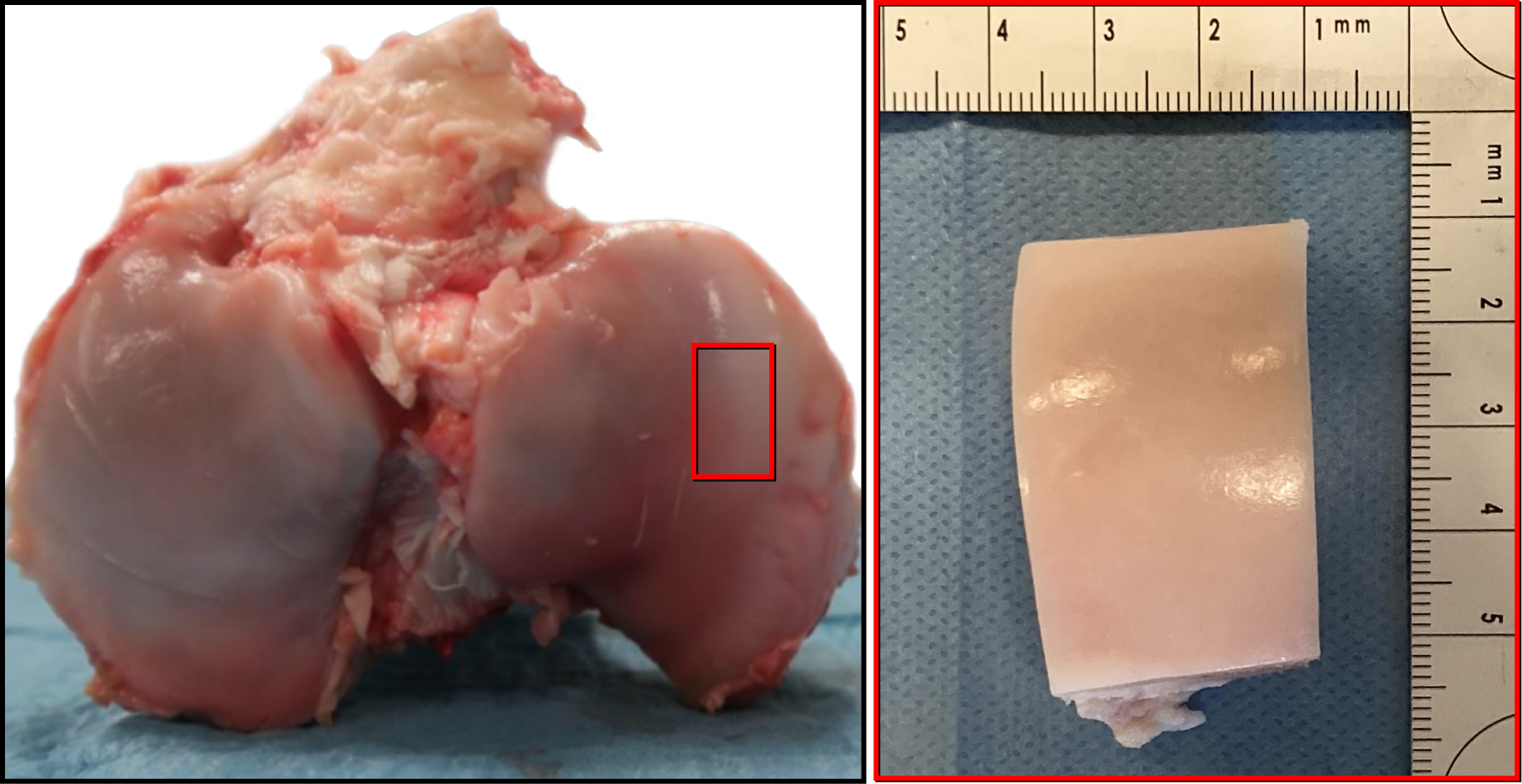

Supplement: Supplementary file 1 [file materials-15-06425-s001.zip › Supplementary Figure S1.tif]

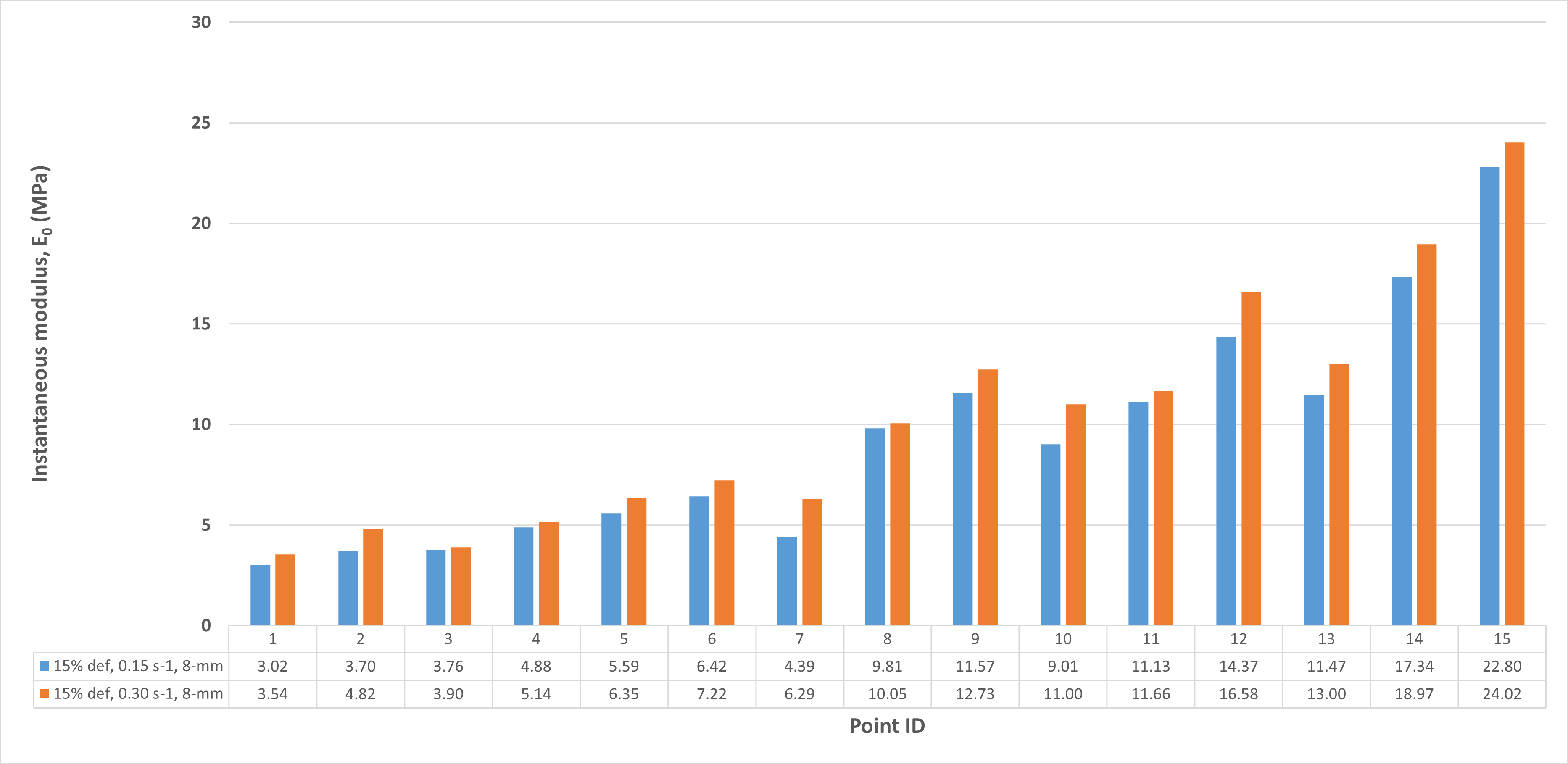

Supplement: Supplementary file 1 [file materials-15-06425-s001.zip › Supplementary Figure S10.tif]

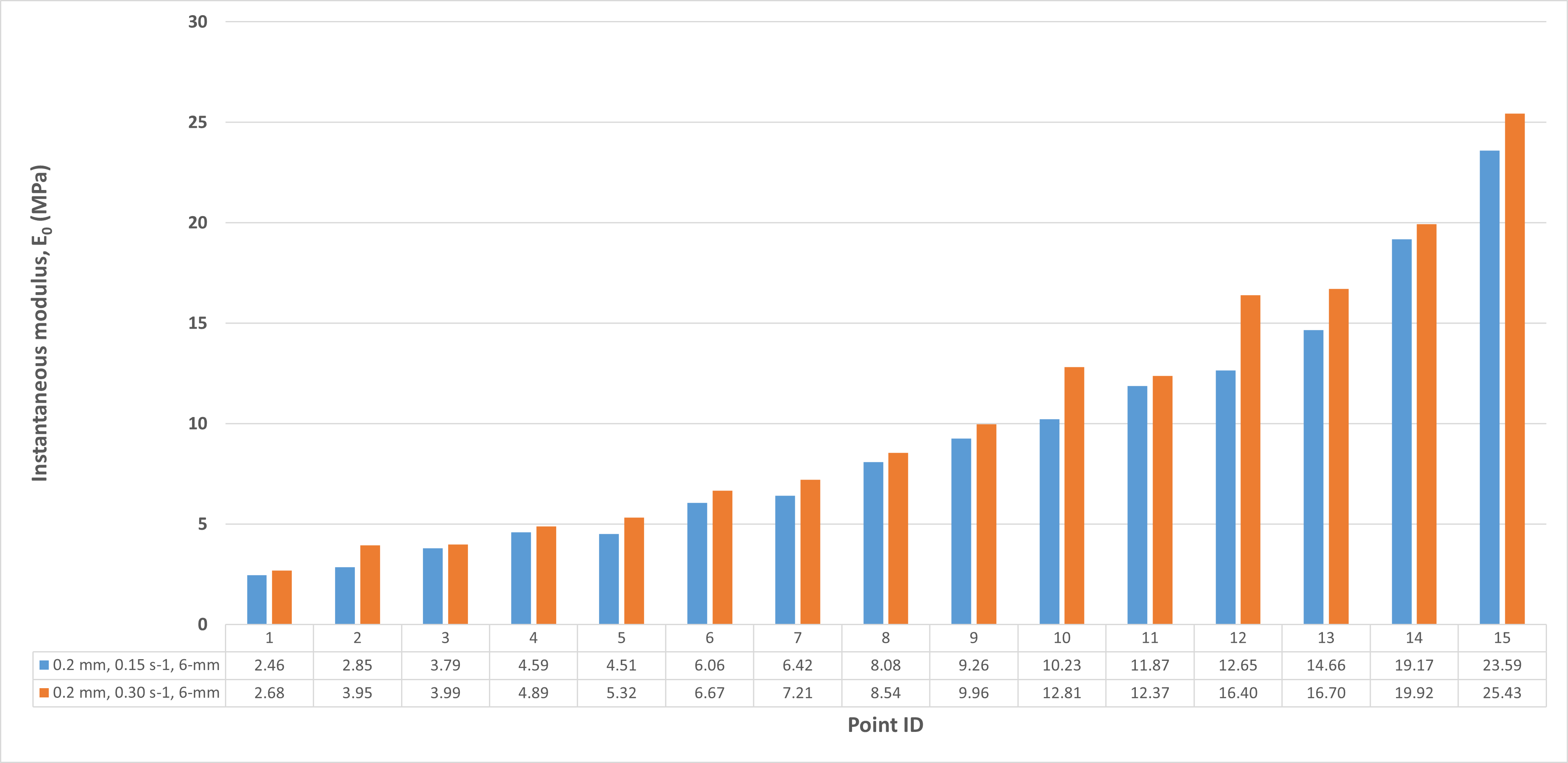

Supplement: Supplementary file 1 [file materials-15-06425-s001.zip › Supplementary Figure S11.tif]

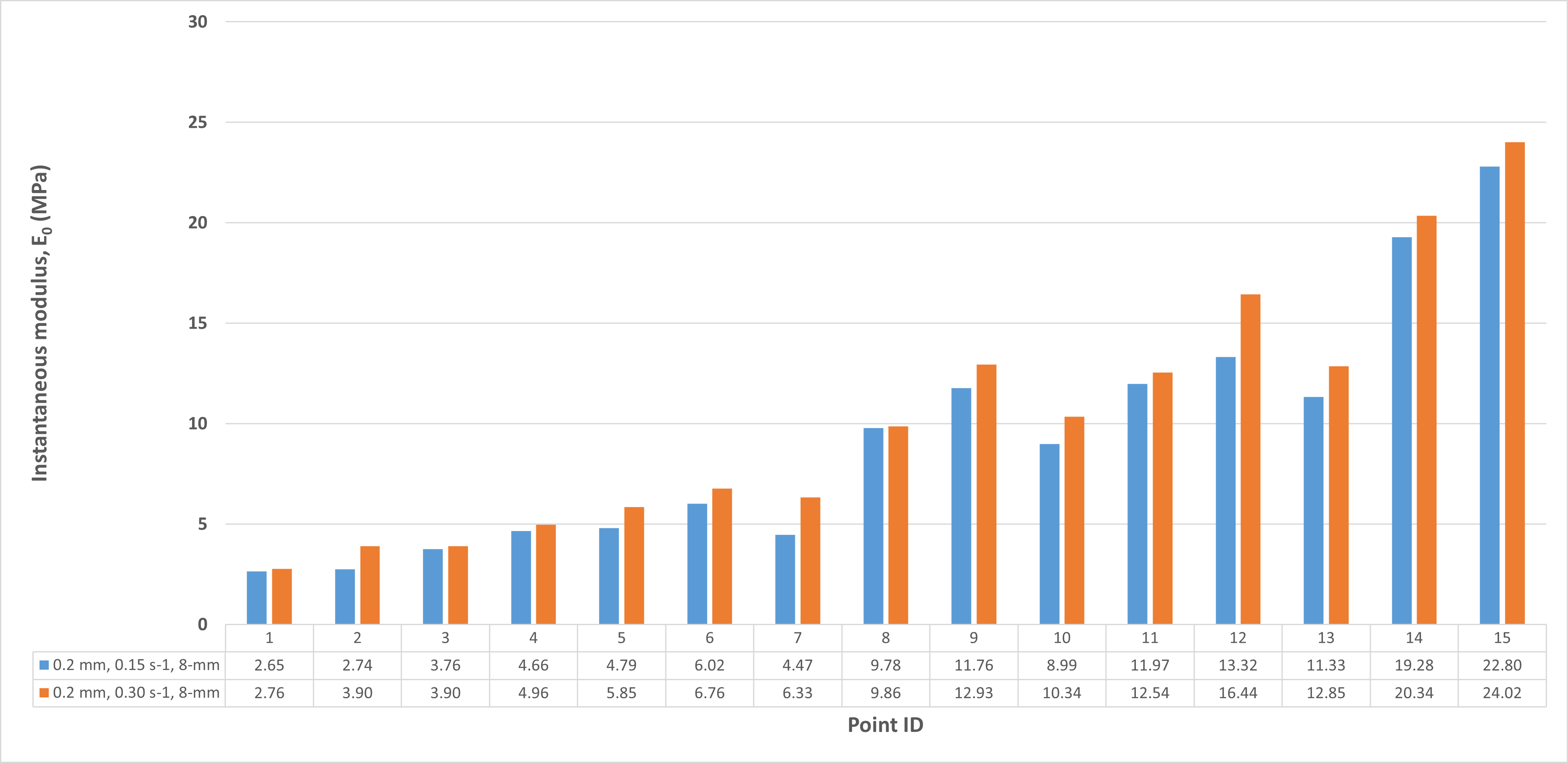

Supplement: Supplementary file 1 [file materials-15-06425-s001.zip › Supplementary Figure S12.tif]

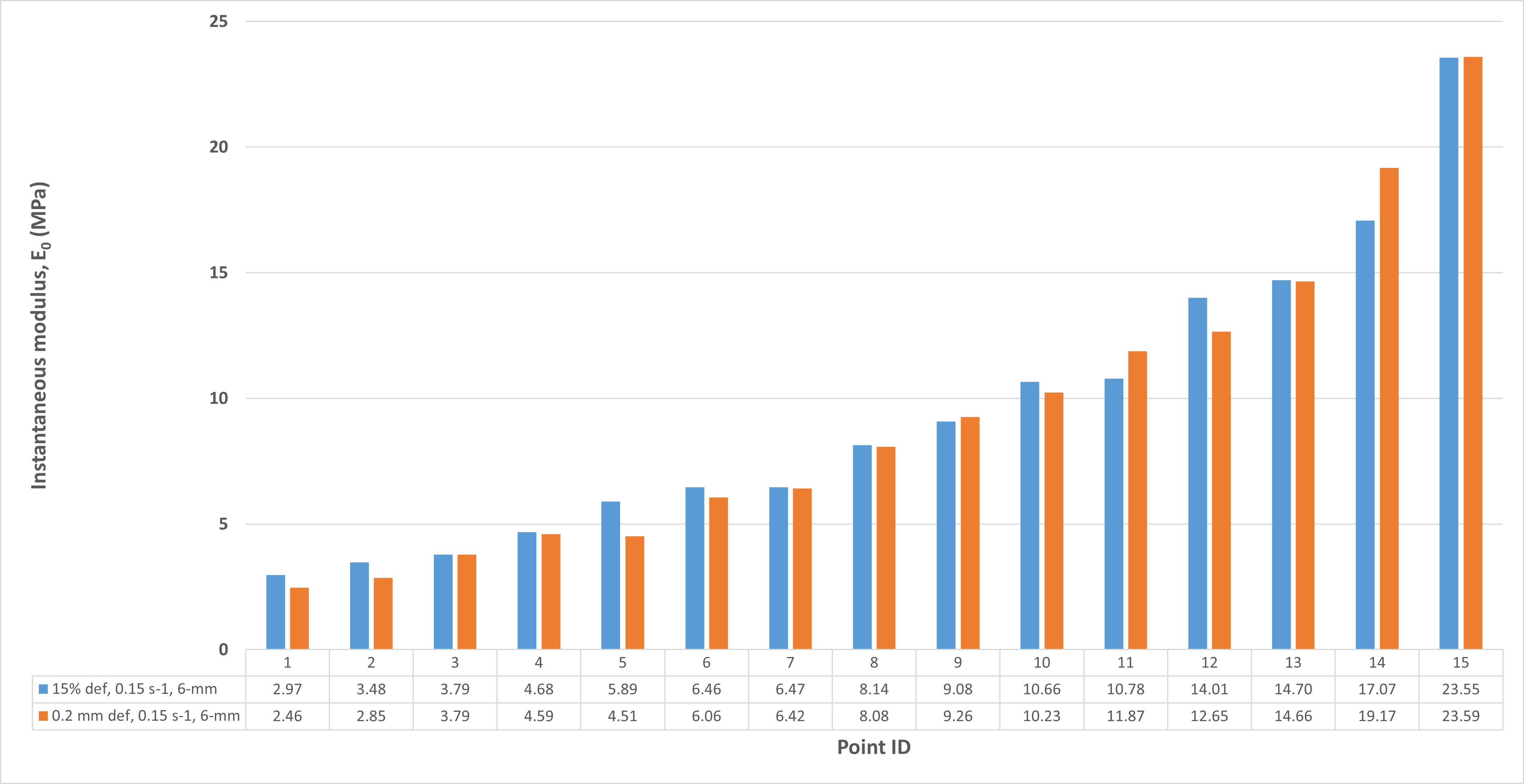

Supplement: Supplementary file 1 [file materials-15-06425-s001.zip › Supplementary Figure S13.tif]

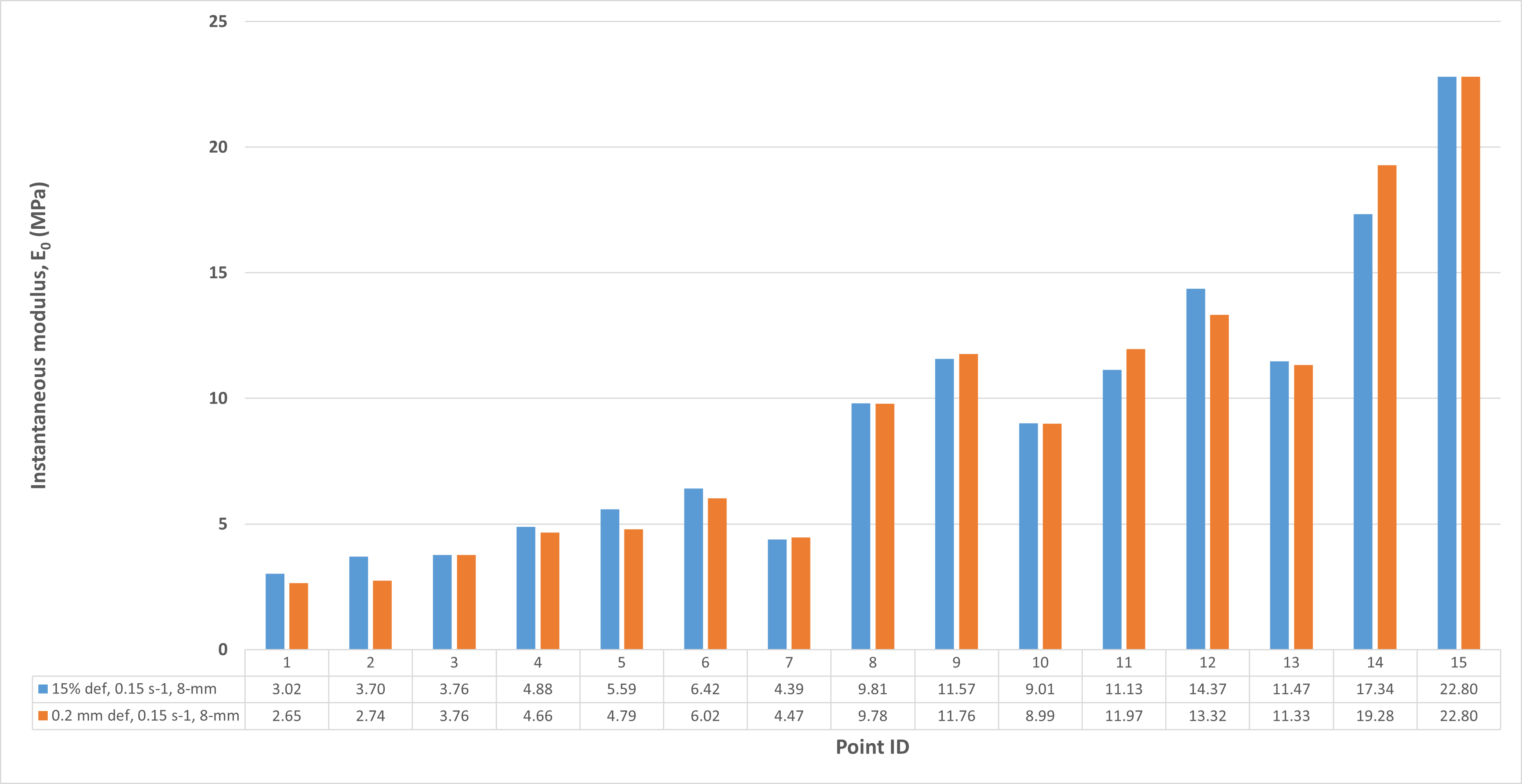

Supplement: Supplementary file 1 [file materials-15-06425-s001.zip › Supplementary Figure S14.tif]

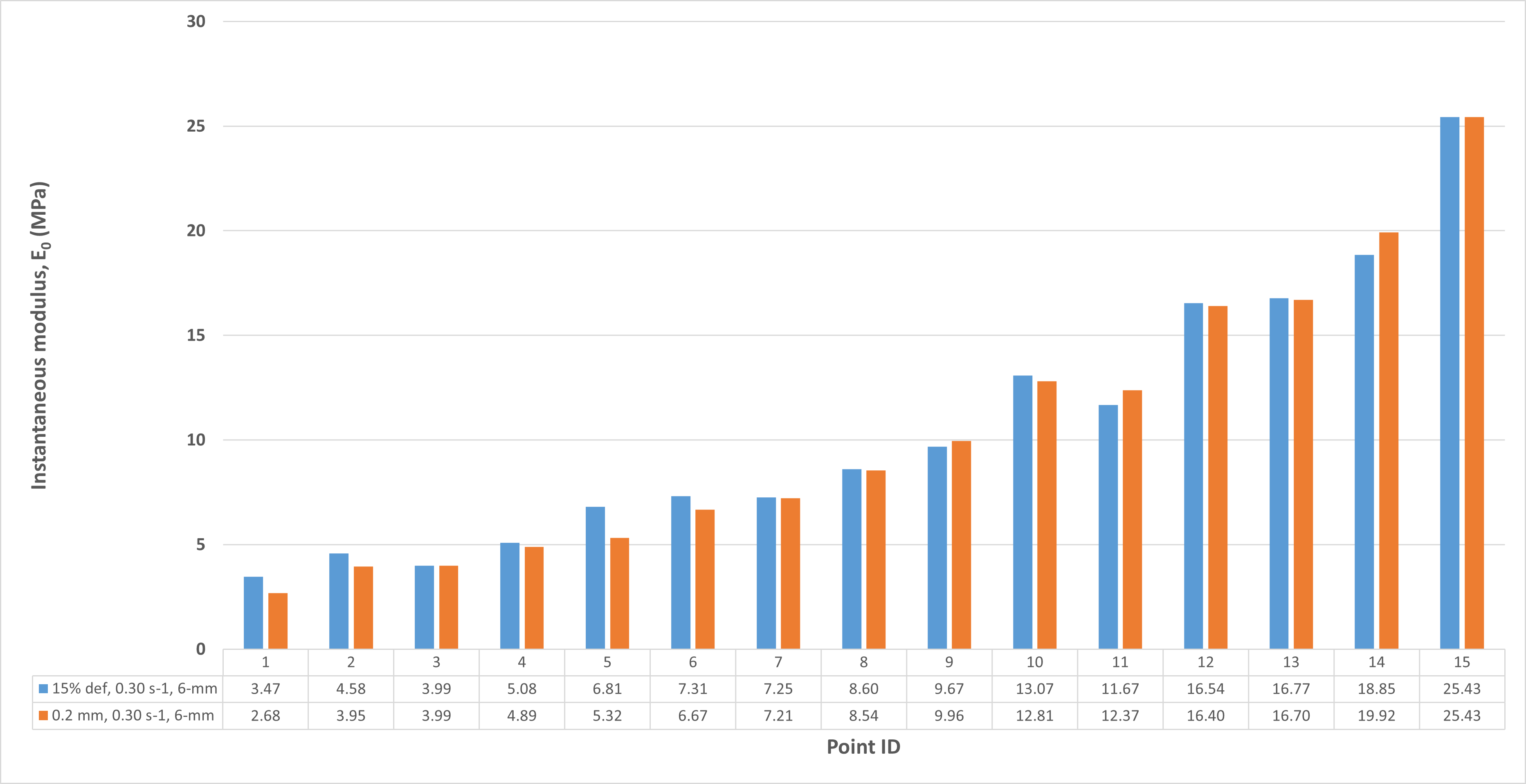

Supplement: Supplementary file 1 [file materials-15-06425-s001.zip › Supplementary Figure S15.tif]

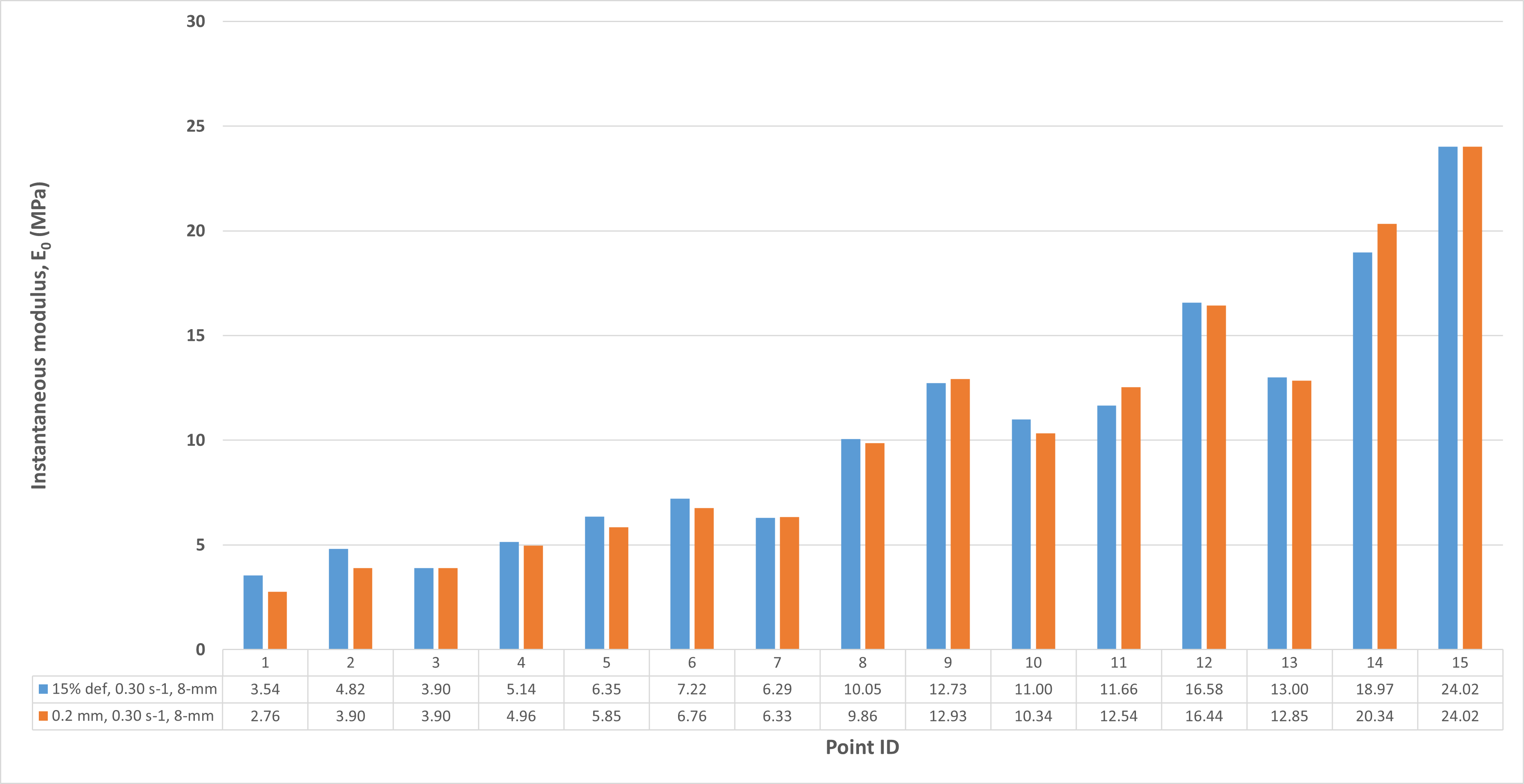

Supplement: Supplementary file 1 [file materials-15-06425-s001.zip › Supplementary Figure S16.tif]

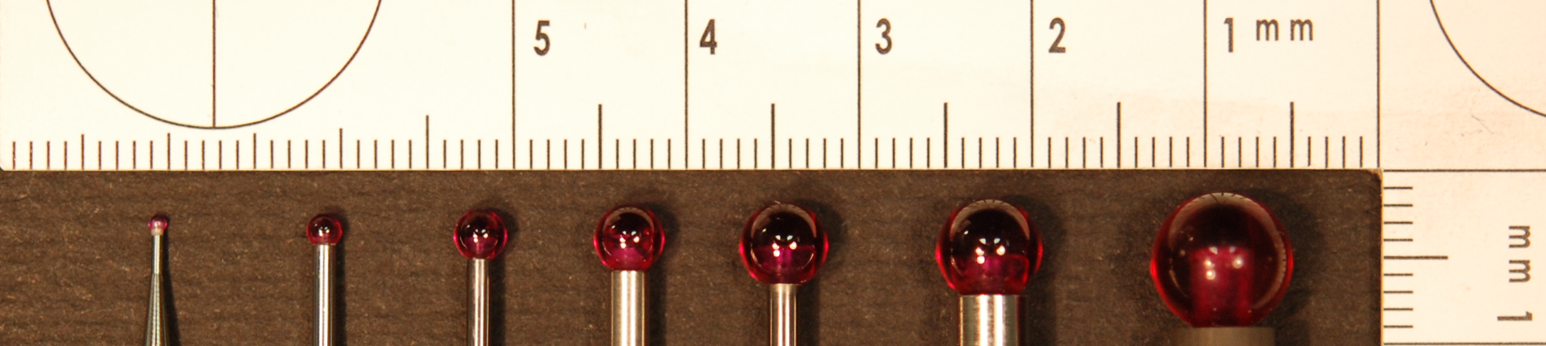

Supplement: Supplementary file 1 [file materials-15-06425-s001.zip › Supplementary Figure S2.png]

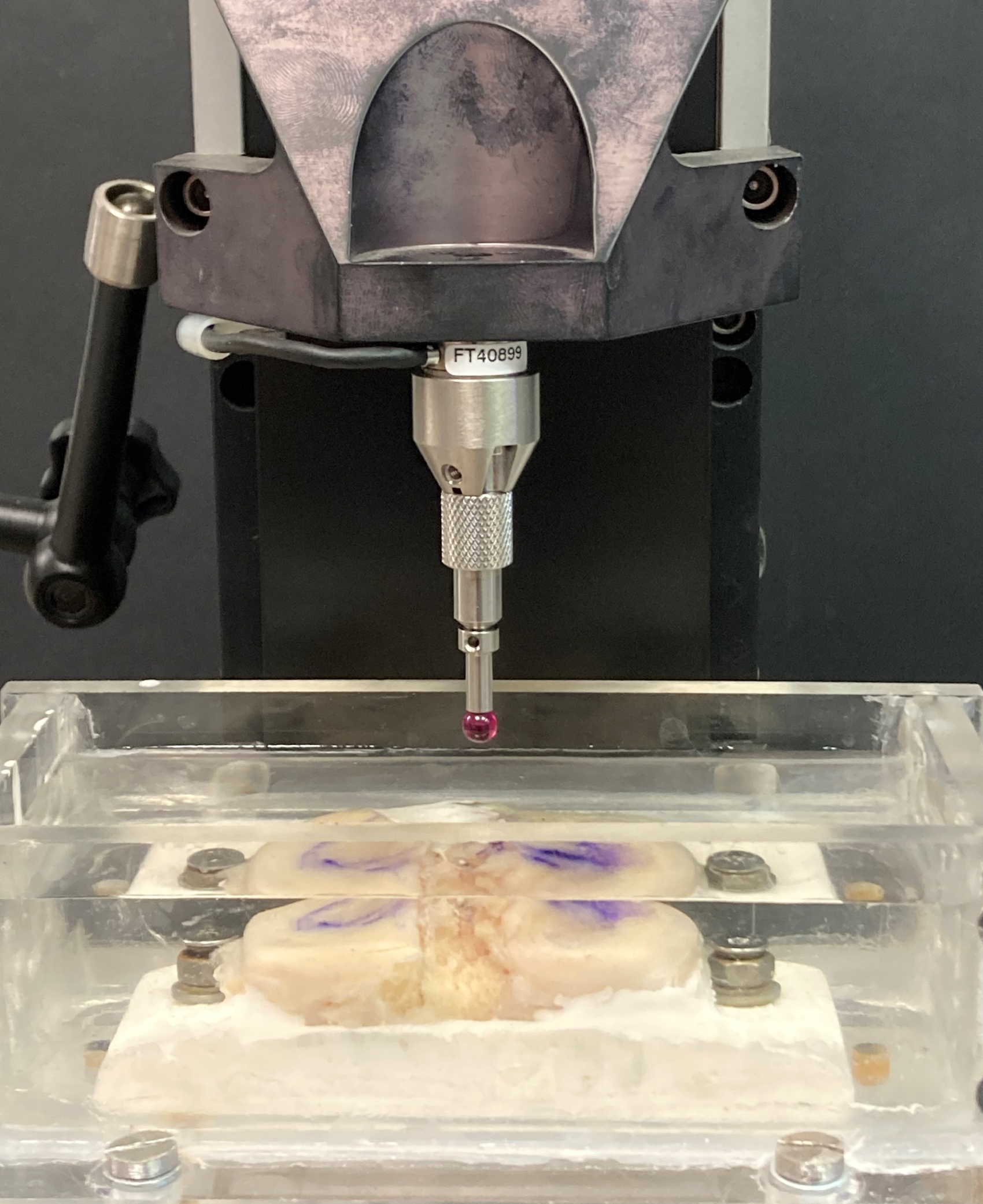

Supplement: Supplementary file 1 [file materials-15-06425-s001.zip › Supplementary Figure S3.png]

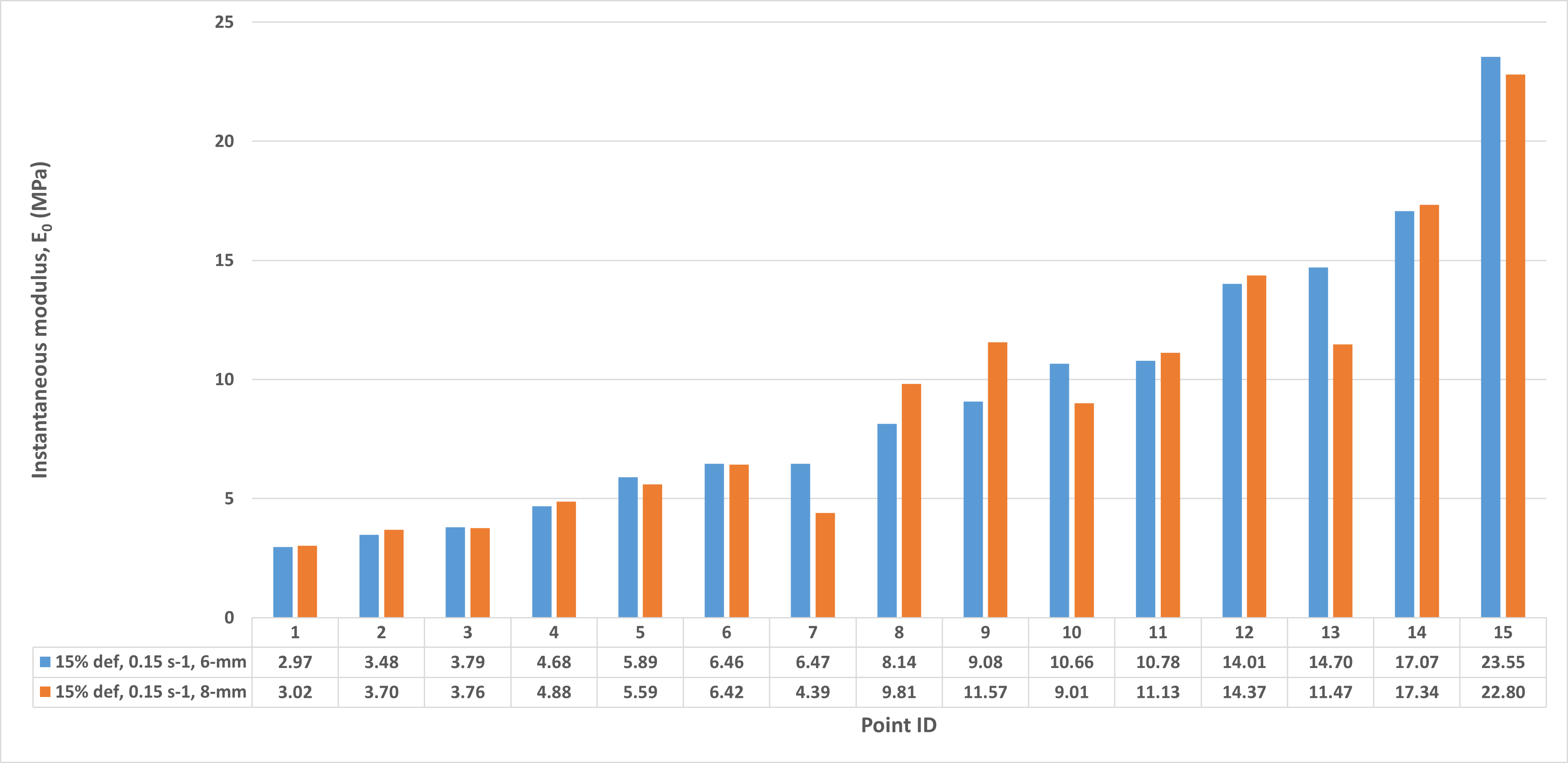

Supplement: Supplementary file 1 [file materials-15-06425-s001.zip › Supplementary Figure S5.tif]

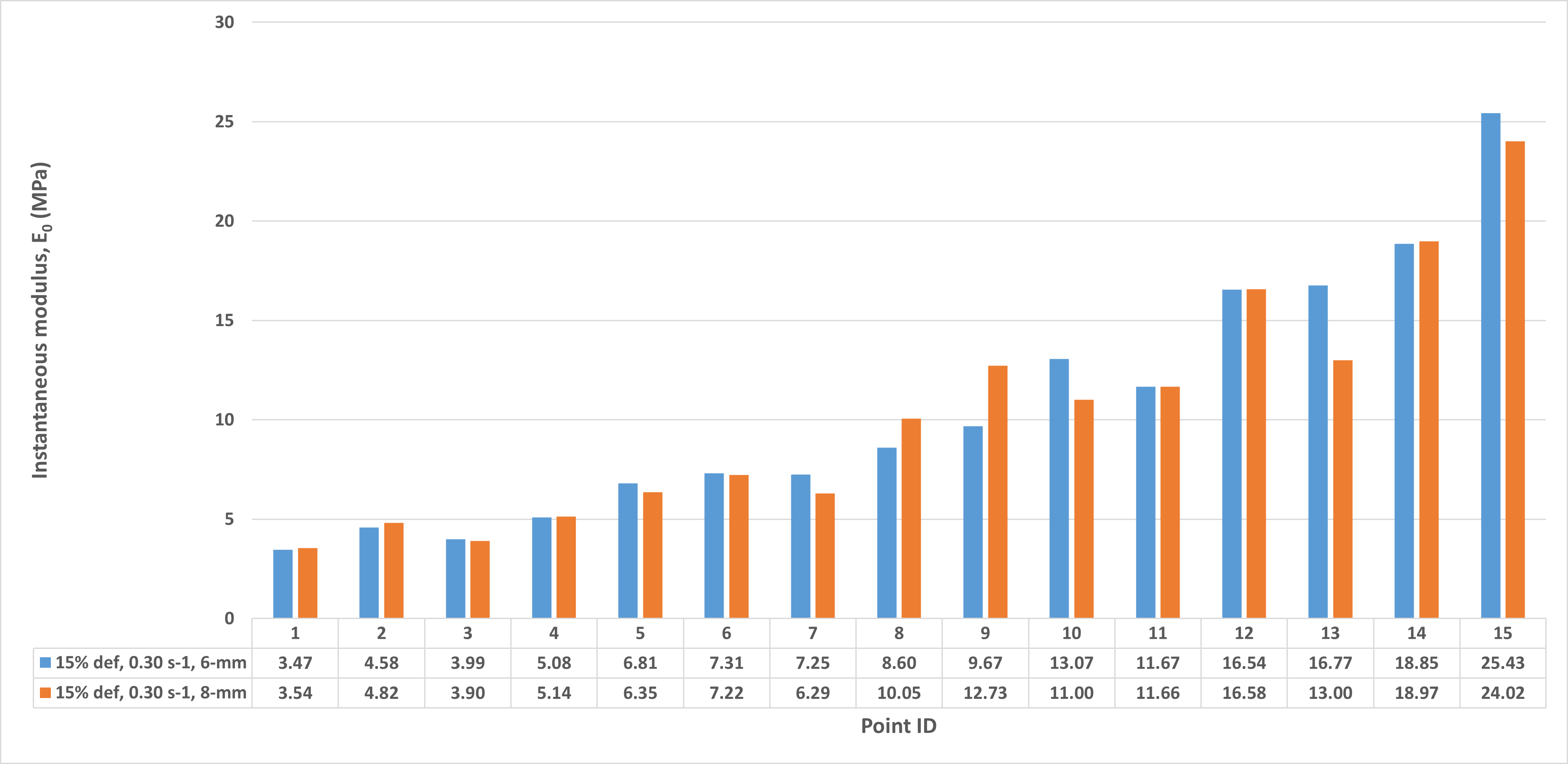

Supplement: Supplementary file 1 [file materials-15-06425-s001.zip › Supplementary Figure S6.tif]

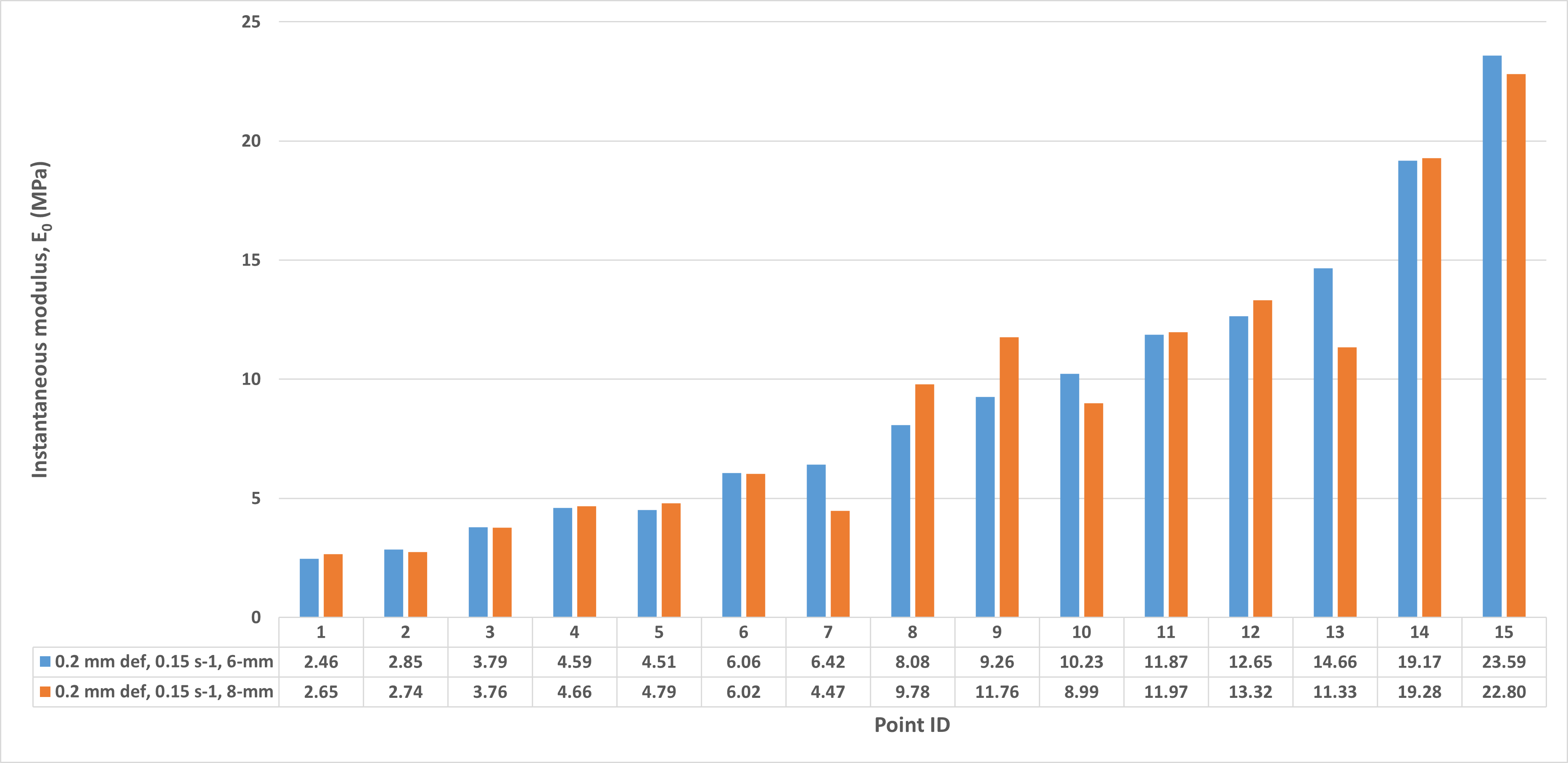

Supplement: Supplementary file 1 [file materials-15-06425-s001.zip › Supplementary Figure S7.tif]

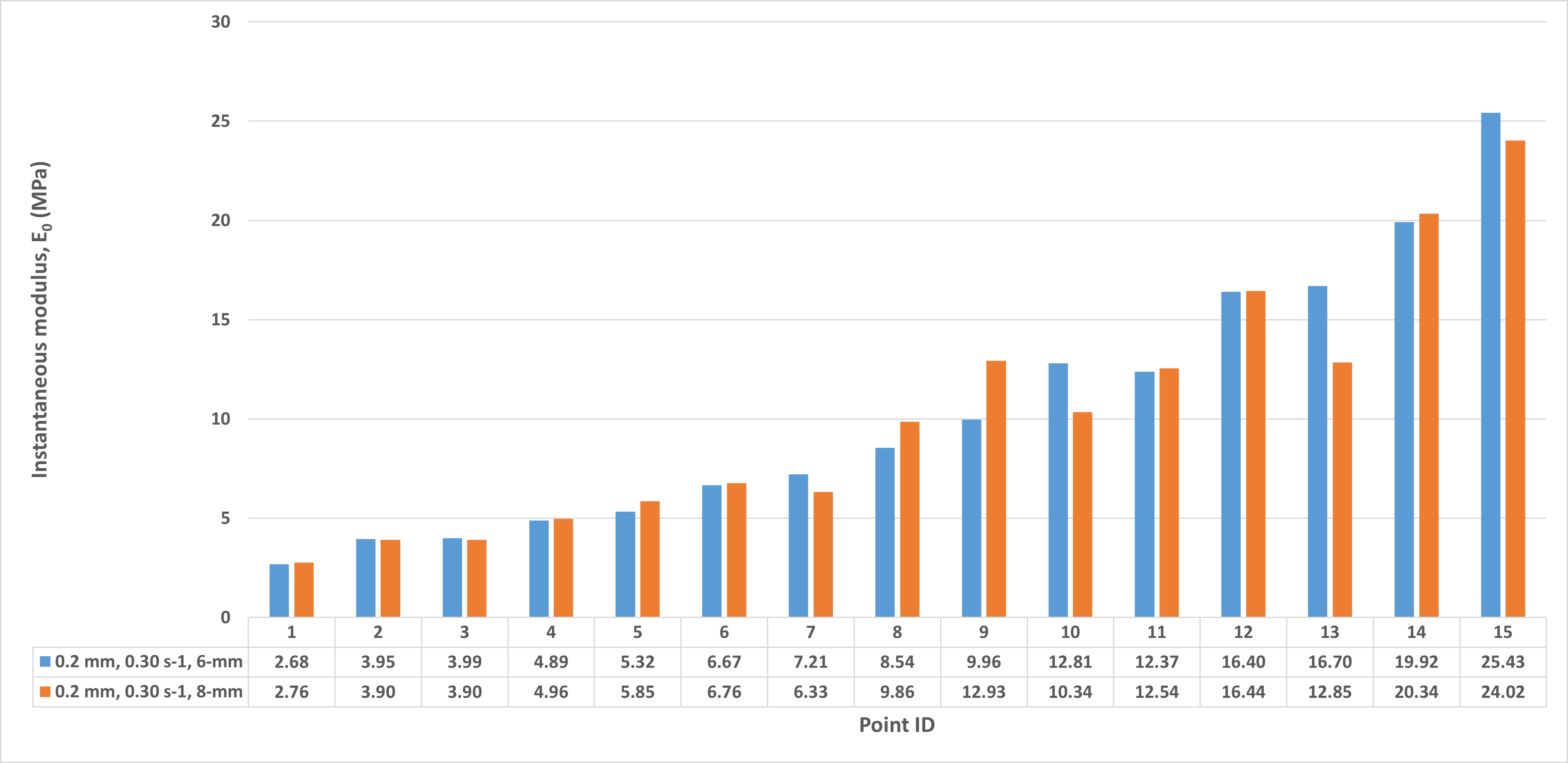

Supplement: Supplementary file 1 [file materials-15-06425-s001.zip › Supplementary Figure S8.tif]

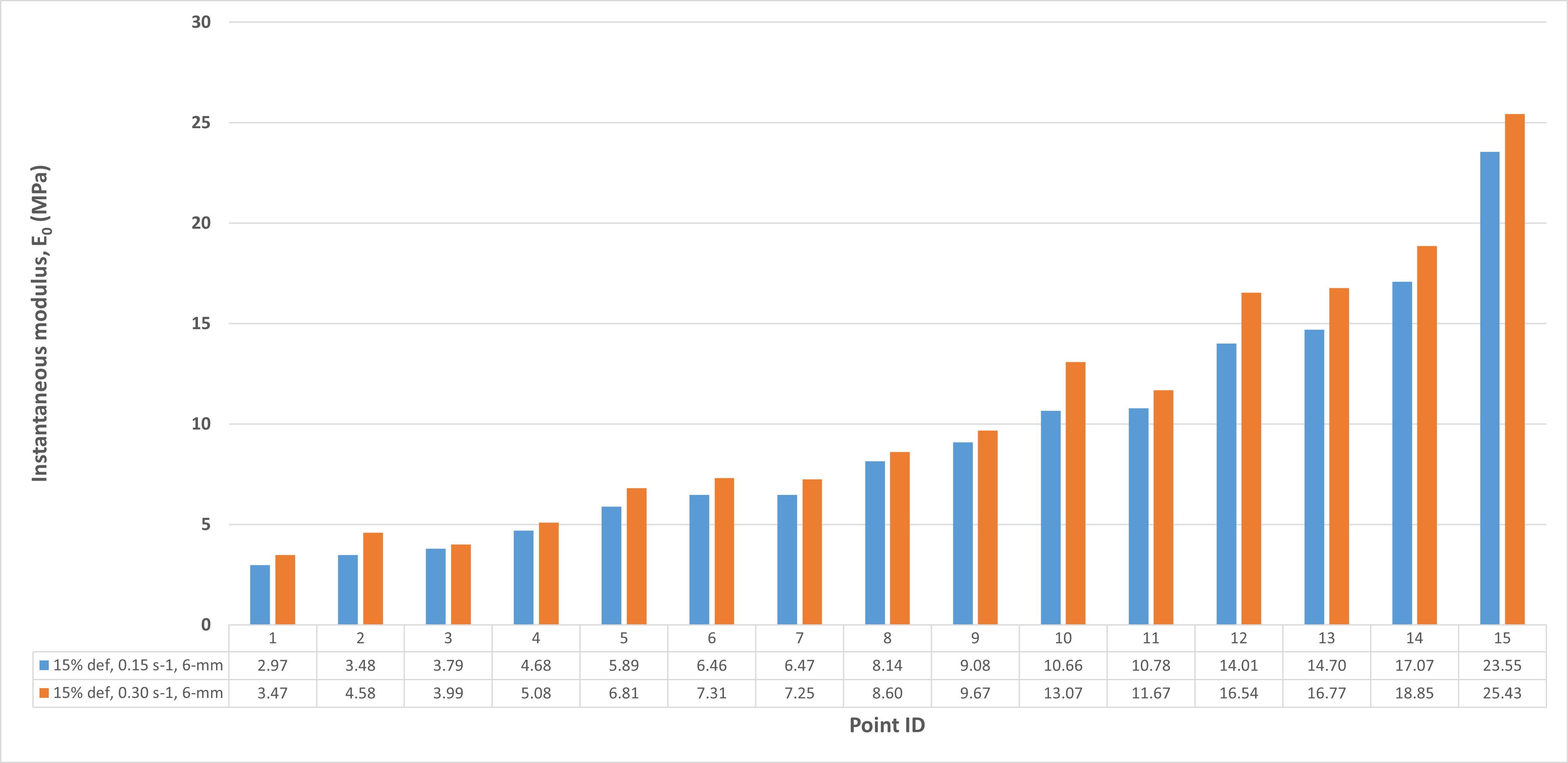

Supplement: Supplementary file 1 [file materials-15-06425-s001.zip › Supplementary Figure S9.tif]
